# Commentary: A Retrospective Study on Using a Novel Single Needle Cone Puncture Approach for the Iodine-125 Seed Brachytherapy in Treating Patients With Thoracic Malignancy

**DOI:** 10.3389/fonc.2022.921080

**Published:** 2022-06-14

**Authors:** Lijuan Zhang, Chenfei Jia, Fenxian Zhang, Enli Chen

**Affiliations:** ^1^ Department of Oncology, Hebei General Hospital, Shijiazhuang, China; ^2^ Department of Oncology, Cangzhou Central Hospital, Cangzhou, China; ^3^ School of Life Science, Shanxi Agricultural University, Jinzhong, China; ^4^ Hebei Seed Diagnosis and Treatment Center, Hebei General Hospital, Shijiazhuang, China

**Keywords:** radioactive iodine-125 brachytherapy, single needle cone puncture, thoracic malignancy, life-threatening symptom, survival

^125^I seed brachytherapy is increasingly used for various types of advanced cancers, and the therapeutic effects are well recognized (1). However, for the treatment of mediastinal tumors, it is difficult to achieve accurate seed distribution and dose distribution. In a recent retrospective study, Li et al. (2) developed a single needle cone puncture method for ^125^ I seed (SNCP-^125^I) brachytherapy for the treatment of thoracic malignancy. The study is especially of great significance in the treatment of patients with lung hilar and mediastinal tumors, which offers an extremely effective therapeutic approach. However, there are still some issues that we expect to discuss with the authors.

First of all, because the only needle used in this technique was repeatedly punctured through tumor lesion, the tumor cells carried by the needle will inevitably be brought to the needle path, which may increase the risk of tumor needle path metastasis. Ryd et al. showed that 10^3^ ~ 10^4^ tumor cells could be implanted in each needle path after the puncture of solid tumors (3). Wang et al. conducted a cytological examination on the tissue smears on the surface of the needle core and needle sheath used in ^125^I seed implantation, and the results showed that the positive rate of tissue smears on the surface of the needle sheath was 5.2% (13/250), and the positive rate of the needle core was 2.8% (7/250). In addition, they showed that the longer the distance of the implanted needle through normal tissue, the more tumor cells will remain in the needle path (4). Therefore, this technique may increase the risk of needle path metastasis.

Secondly, SNCP-^125^I not only is difficult to operate but also has high technical requirements and a long learning curve, which prevents its broad application in the clinical practice. In recent years, the technology of three-dimensional (3D) printing template-guided ^125^I seed implantation has developed rapidly, which can achieve a more accurate distribution of the seed and dose while greatly reducing operation difficulty (5, 6). Ji et al. (7) treated 22 patients with a thoracic malignant tumor with a 3D printing coplanar coordinate template (3D-PCCT) -assisted CT-guided ^125^I seed implantation. There were 2 patients whose tumor was located in the mediastinum. All operations were successfully performed, and the distribution of the seed and dose was consistent with the preoperative plan. These results indicate that 3D-PCCT-assisted ^125^I seed implantation is safe and effective in the treatment of mediastinal tumors. Lv et al. (8) applied coplanar template (CPT)-assisted ^125^I seed implantation to treat 32 patients with advanced lung cancer with mediastinal lymph node metastasis in the 4R group. The results showed that all patients successfully completed seed implantation, and the postoperative dose distribution and dose of organs at risk were consistent with the preoperative plan. In addition, the effective rate was 84.37% 6 months after the operation, and no lung radiation injury, large vessel injury, bleeding, and other serious complications were found during the follow-up. It is suggested that template-assisted CT-guided ^125^I seed implantation for mediastinal lymph node metastasis can better achieve the preoperative plan and avoid important blood vessel and organ damage, which is an accurate, effective, and safe treatment method. Therefore, the SNCP-^125^I technique should be preferred when there are few puncture paths, and the template guidance method should be preferred in other cases.

In conclusion, the SNCP-^125^I technique may increase the risk of needle path metastasis due to repeated tumor lesion puncture. If SNCP-^125^I is to be administered, it is necessary to improve the puncture level of the operator, optimize the preoperative treatment plan, and take corresponding preventive measures during the operation, as well as active anti-tumor treatment and regular follow-ups after the operation. At last, template-assisted ^125^I seed implantation should be preferred in some cases.

## Author Contributions

LZ, CJ, FZ, and EC wrote the letter. All authors contributed to the article and approved the submitted version.

## Conflict of Interest 

The authors declare that the research was conducted in the absence of any commercial or financial relationships that could be construed as a potential conflict of interest.

## Publisher’s Note

All claims expressed in this article are solely those of the authors and do not necessarily represent those of their affiliated organizations, or those of the publisher, the editors and the reviewers. Any product that may be evaluated in this article, or claim that may be made by its manufacturer, is not guaranteed or endorsed by the publisher.
